# *rac*-5-Acetyl-6-hy­droxy-3,6-dimethyl-4-phenyl-2*H*-4,5,6,7-tetra­hydro­indazol-1-ium chloride

**DOI:** 10.1107/S1600536810024281

**Published:** 2010-06-26

**Authors:** Abel M. Maharramov, Arif I. Ismiyev, Bahruz A. Rashidov, Rizvan K. Askerov, Victor N. Khrustalev

**Affiliations:** a Baku State University, Z. Khalilov St. 23, Baku, AZ-1148, Azerbaijan; b X-Ray Structural Centre, A.N.Nesmeyanov Institute of Organoelement Compounds, Russian Academy of Sciences, 28 Vavilov St., B-334, Moscow 119991, Russian Federation

## Abstract

The structure of the title compound, C_17_H_21_N_2_O_2_^+^·Cl^−^, is of inter­est with respect to its biological activity. The title compound comprises an organic cation and a chloride anion in the asymmetric unit. The positive charge is localized in a pyrazole moiety forming a pyrazolium cation. The structure displays inter­molecular O—H⋯Cl and N—H⋯Cl hydrogen bonding.

## Related literature

For general background, see: Raptis *et al.* (1993 ▶); Rabe (1904 ▶).
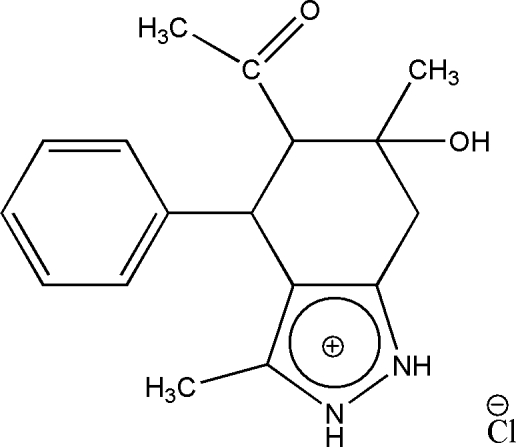


## Experimental

### 

#### Crystal data

C_17_H_21_N_2_O_2_^+^·Cl^−^
*M* *_r_* = 320.81
Triclinic, 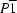

*a* = 6.9661 (3) Å
*b* = 8.3527 (4) Å
*c* = 15.6739 (7) Å
α = 88.145 (1)°
β = 87.385 (1)°
γ = 67.882 (1)°
*V* = 843.89 (7) Å^3^
*Z* = 2
Mo *K*α radiation
μ = 0.24 mm^−1^
*T* = 296 K
0.30 × 0.30 × 0.20 mm

#### Data collection

Bruker APEXII CCD diffractometer
Absorption correction: multi-scan (*SADABS*; Sheldrick, 2003 ▶) *T*_min_ = 0.933, *T*_max_ = 0.955
9861 measured reflections
4188 independent reflections
3279 reflections with *I* > 2σ(*I*)
*R*_int_ = 0.015

#### Refinement

*R*[*F* ^2^ > 2σ(*F* ^2^)] = 0.043
*wR*(*F* ^2^) = 0.130
*S* = 1.00
4188 reflections
199 parameters
H-atom parameters constrained
Δρ_max_ = 0.31 e Å^−3^
Δρ_min_ = −0.23 e Å^−3^

### 

Data collection: *APEX2* (Bruker, 2005 ▶); cell refinement: *SAINT-Plus* (Bruker, 2001 ▶); data reduction: *SAINT-Plus*; program(s) used to solve structure: *SHELXTL* (Sheldrick, 2008 ▶); program(s) used to refine structure: *SHELXTL*; molecular graphics: *SHELXTL*; software used to prepare material for publication: *SHELXTL*.

## Supplementary Material

Crystal structure: contains datablocks global, I. DOI: 10.1107/S1600536810024281/kp2265sup1.cif

Structure factors: contains datablocks I. DOI: 10.1107/S1600536810024281/kp2265Isup2.hkl

Additional supplementary materials:  crystallographic information; 3D view; checkCIF report

## Figures and Tables

**Table 1:** Hydrogen-bond geometry (Å, °)

*D*—H⋯*A*	*D*—H	H⋯*A*	*D*⋯*A*	*D*—H⋯*A*
O1—H1*A*⋯Cl	0.82	2.39	3.2110 (14)	176
N2—H2*B*⋯Cl	0.86	2.21	3.0620 (14)	171
N1—H1*B*⋯Cl	0.86	2.25	3.0280 (15)	150

**Fig. 1.**
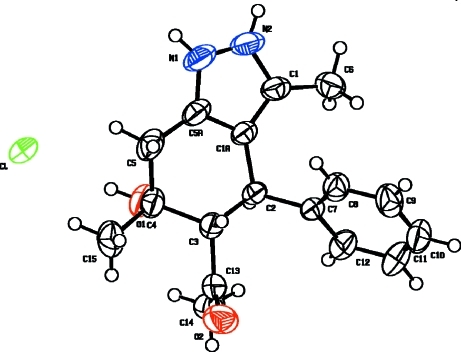
The molecular structure of the title compound, with the atomic numbering scheme. Displacement ellipsoids were drawn at the 30% probability level.

**Fig. 2.**
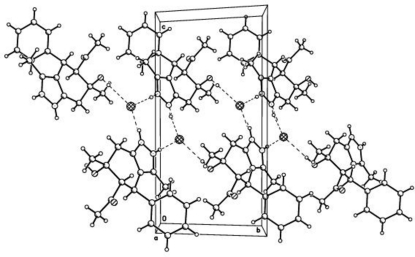
A hydrogen-bonded (dashed lines) ribbon in the title compound. H atoms not involved in hydrogen bonding have been omitted for clarity.

## supplementary crystallographic information

### Comment

(*rac*)-5-Acetyl-6-hydroxy-3,6-dimethyl-4-phenyl-2*H*-4,5,6,7- tetrahyroindazolium chloride (I) have good antibacterial and biological properties. We have synthesised the title compound, (I), and its structure is reported here (Fig. 1). The two [(C2(*R*),C4(*R*)] of three stereogenic centres of tetrahydroindazole moiety are of the same chirality. As the crystal crystallises in the centrosymmetric space group, the racemate (1:1) is present. The crystal structure involves O—H···Cl, and N—H···C intermolecular hydrogen bonds (Table 1 and Fig. 2).

### Experimental

2,4-Diacetyl-5-hydroxy-5-methyl-3-phenylcyclohexanon (20 mmol)and hydrazine hydrate (20 mmol) were dissolved in 20 ml ethanol. The mixture was stirred at 340 K within 10 h. Through suspension in absolute toluene a flow of dry gaseous hydrogen chloride was used at 278-283 K. From a solution a white solid was obtained. A crude product was filtered and washed with ethanol. Then, the crude product was dissolved in ethanol (50 ml) and recrystallised to yield colourless block-shaped crystals of (I).

### Refinement

The hydrogen atoms of the OH and NH-groups of the molecule (I) were localized in a difference-Fourier map and included in the refinement with fixed positional and isotropic displacement parameters [*U*_iso_ (H) = 1.5*U*_eq_(C) for CH_3_-group and *U*_iso_(H) = 1.2*U*_eq_(N) for amino groups]. The other hydrogen atoms were placed in calculated positions with and refined in the riding model with fixed isotropic displacement parameters [*U*_iso_(H) = 1.2*U*_eq_(C)]. 24 reflections, with experimentally observed *F*^2 ^deviating significantly from the theoretically calculated *F*^2^, were omitted from the refinement. Moreover, 76 reflections were not measured because the angle limits.

### Crystal data

**Table:**

C_17_H_21_N_2_O_2_^+^·Cl^−^	*Z* = 2
*M**_r_* = 320.81	*F*(000) = 340
Triclinic, *P*1	*D*_x_ = 1.263 Mg m^−^^3^
Hall symbol: -P 1	Mo *K*α radiation, λ = 0.71073 Å
*a* = 6.9661 (3) Å	Cell parameters from 4494 reflections
*b* = 8.3527 (4) Å	θ = 2.6–28.3°
*c* = 15.6739 (7) Å	µ = 0.24 mm^−^^1^
α = 88.145 (1)°	*T* = 296 K
β = 87.385 (1)°	Prism, colourless
γ = 67.882 (1)°	0.30 × 0.30 × 0.20 mm
*V* = 843.89 (7) Å^3^	

### Data collection

**Table:**

Bruker APEXII CCD diffractometer	4188 independent reflections
Radiation source: fine-focus sealed tube	3279 reflections with *I* > 2σ(*I*)
graphite	*R*_int_ = 0.015
φ and ω scans	θ_max_ = 28.4°, θ_min_ = 2.6°
Absorption correction: multi-scan (*SADABS*; Sheldrick, 2003)	*h* = −9→9
*T*_min_ = 0.933, *T*_max_ = 0.955	*k* = −11→11
9861 measured reflections	*l* = −20→20

### Refinement

**Table:**

Refinement on *F*^2^	Primary atom site location: structure-invariant direct methods
Least-squares matrix: full	Secondary atom site location: difference Fourier map
*R*[*F*^2^ > 2σ(*F*^2^)] = 0.043	Hydrogen site location: difference Fourier map
*wR*(*F*^2^) = 0.130	H-atom parameters constrained
*S* = 1.00	*w* = 1/[σ^2^(*F*_o_^2^) + (0.0702*P*)^2^ + 0.1788*P*] where *P* = (*F*_o_^2^ + 2*F*_c_^2^)/3
4188 reflections	(Δ/σ)_max_ < 0.001
199 parameters	Δρ_max_ = 0.31 e Å^−^^3^
0 restraints	Δρ_min_ = −0.23 e Å^−^^3^

### Special details

**Table:**

Geometry. All e.s.d.'s (except the e.s.d. in the dihedral angle between two l.s. planes) are estimated using the full covariance matrix. The cell e.s.d.'s are taken into account individually in the estimation of e.s.d.'s in distances, angles and torsion angles; correlations between e.s.d.'s in cell parameters are only used when they are defined by crystal symmetry. An approximate (isotropic) treatment of cell e.s.d.'s is used for estimating e.s.d.'s involving l.s. planes.
Refinement. Refinement of *F*^2^ against ALL reflections. The weighted *R*-factor *wR* and goodness of fit *S* are based on *F*^2^, conventional *R*-factors *R* are based on *F*, with *F* set to zero for negative *F*^2^. The threshold expression of *F*^2^ > σ(*F*^2^) is used only for calculating *R*-factors(gt) *etc*. and is not relevant to the choice of reflections for refinement. *R*-factors based on *F*^2^ are statistically about twice as large as those based on *F*, and *R*- factors based on ALL data will be even larger.

### Fractional atomic coordinates and isotropic or equivalent isotropic displacement parameters (Å^2^)

**Table:**

	*x*	*y*	*z*	*U*_iso_*/*U*_eq_	
Cl	0.79637 (7)	0.21601 (6)	0.42366 (3)	0.06850 (18)	
O1	0.82690 (19)	0.50419 (14)	0.28900 (7)	0.0544 (3)	
H1A	0.8256	0.4295	0.3240	0.082*	
C2	0.6910 (2)	0.84494 (17)	0.21715 (8)	0.0370 (3)	
H2A	0.6654	0.7634	0.1801	0.044*	
C7	0.6848 (2)	1.00157 (18)	0.16319 (9)	0.0403 (3)	
C3	0.9075 (2)	0.75279 (17)	0.25585 (9)	0.0407 (3)	
H3A	0.9415	0.8418	0.2835	0.049*	
C1A	0.5285 (2)	0.89218 (18)	0.28808 (9)	0.0408 (3)	
O2	1.2259 (2)	0.71271 (18)	0.18122 (10)	0.0707 (4)	
N2	0.2393 (2)	0.9911 (2)	0.36527 (10)	0.0608 (4)	
H2B	0.1114	1.0441	0.3808	0.073*	
C1	0.3197 (2)	0.99656 (19)	0.28640 (10)	0.0469 (3)	
C13	1.0750 (2)	0.67523 (19)	0.18596 (10)	0.0475 (3)	
C8	0.6566 (2)	1.1566 (2)	0.20107 (11)	0.0484 (3)	
H8A	0.6442	1.1642	0.2603	0.058*	
C5A	0.5664 (3)	0.8269 (2)	0.37032 (9)	0.0497 (4)	
N1	0.3884 (3)	0.8905 (2)	0.41623 (9)	0.0626 (4)	
H1B	0.3722	0.8703	0.4697	0.075*	
C6	0.1911 (2)	1.0995 (2)	0.21689 (12)	0.0557 (4)	
H6A	0.0508	1.1562	0.2381	0.084*	
H6B	0.1942	1.0247	0.1712	0.084*	
H6C	0.2445	1.1845	0.1962	0.084*	
C4	0.9097 (3)	0.6160 (2)	0.32611 (9)	0.0483 (3)	
C12	0.7041 (3)	0.9940 (3)	0.07526 (11)	0.0643 (5)	
H12A	0.7216	0.8916	0.0483	0.077*	
C9	0.6467 (3)	1.3007 (2)	0.15225 (14)	0.0623 (5)	
H9A	0.6254	1.4045	0.1787	0.075*	
C14	1.0503 (3)	0.5535 (2)	0.12344 (12)	0.0611 (4)	
H14A	1.1675	0.5174	0.0839	0.092*	
H14B	0.9259	0.6107	0.0928	0.092*	
H14C	1.0416	0.4543	0.1535	0.092*	
C5	0.7689 (3)	0.7091 (2)	0.40209 (10)	0.0577 (4)	
H5A	0.7487	0.6250	0.4419	0.069*	
H5B	0.8335	0.7746	0.4318	0.069*	
C15	1.1284 (3)	0.5161 (3)	0.35650 (13)	0.0697 (5)	
H15A	1.1245	0.4336	0.3999	0.104*	
H15B	1.1824	0.5952	0.3795	0.104*	
H15C	1.2160	0.4568	0.3092	0.104*	
C11	0.6976 (4)	1.1385 (3)	0.02648 (13)	0.0857 (7)	
H11A	0.7135	1.1313	−0.0327	0.103*	
C10	0.6680 (3)	1.2903 (3)	0.06526 (15)	0.0771 (6)	
H10A	0.6624	1.3867	0.0325	0.092*	

### Atomic displacement parameters (Å^2^)

**Table:**

	*U*^11^	*U*^22^	*U*^33^	*U*^12^	*U*^13^	*U*^23^
Cl	0.0681 (3)	0.0699 (3)	0.0529 (2)	−0.0135 (2)	0.0232 (2)	0.0174 (2)
O1	0.0784 (8)	0.0446 (6)	0.0443 (6)	−0.0286 (6)	−0.0005 (5)	0.0089 (4)
C2	0.0406 (7)	0.0392 (6)	0.0305 (6)	−0.0150 (5)	0.0049 (5)	0.0008 (5)
C7	0.0331 (6)	0.0466 (7)	0.0376 (6)	−0.0120 (5)	0.0024 (5)	0.0094 (5)
C3	0.0458 (7)	0.0372 (6)	0.0379 (6)	−0.0148 (6)	0.0001 (5)	0.0027 (5)
C1A	0.0477 (7)	0.0400 (7)	0.0371 (6)	−0.0202 (6)	0.0090 (5)	−0.0008 (5)
O2	0.0494 (7)	0.0660 (8)	0.0957 (10)	−0.0224 (6)	0.0128 (6)	0.0015 (7)
N2	0.0598 (8)	0.0617 (8)	0.0610 (8)	−0.0257 (7)	0.0290 (7)	−0.0075 (7)
C1	0.0488 (8)	0.0423 (7)	0.0520 (8)	−0.0216 (6)	0.0165 (6)	−0.0059 (6)
C13	0.0442 (7)	0.0380 (7)	0.0539 (8)	−0.0096 (6)	0.0047 (6)	0.0090 (6)
C8	0.0455 (8)	0.0493 (8)	0.0530 (8)	−0.0212 (6)	−0.0043 (6)	0.0093 (6)
C5A	0.0667 (10)	0.0497 (8)	0.0354 (7)	−0.0265 (7)	0.0129 (6)	−0.0014 (6)
N1	0.0789 (10)	0.0658 (9)	0.0437 (7)	−0.0307 (8)	0.0246 (7)	−0.0027 (6)
C6	0.0420 (8)	0.0487 (8)	0.0718 (11)	−0.0128 (7)	0.0071 (7)	0.0005 (7)
C4	0.0620 (9)	0.0437 (7)	0.0372 (7)	−0.0179 (7)	−0.0044 (6)	0.0075 (6)
C12	0.0766 (12)	0.0636 (10)	0.0398 (8)	−0.0138 (9)	0.0079 (8)	0.0095 (7)
C9	0.0492 (9)	0.0508 (9)	0.0895 (13)	−0.0225 (7)	−0.0095 (8)	0.0199 (9)
C14	0.0675 (11)	0.0518 (9)	0.0554 (9)	−0.0145 (8)	0.0176 (8)	−0.0058 (7)
C5	0.0796 (11)	0.0598 (10)	0.0326 (7)	−0.0256 (9)	0.0001 (7)	0.0065 (6)
C15	0.0735 (12)	0.0644 (11)	0.0615 (11)	−0.0147 (9)	−0.0182 (9)	0.0181 (9)
C11	0.0979 (16)	0.0927 (16)	0.0494 (10)	−0.0205 (13)	0.0106 (10)	0.0316 (11)
C10	0.0620 (11)	0.0730 (13)	0.0880 (14)	−0.0198 (10)	−0.0012 (10)	0.0454 (12)

### Geometric parameters (Å, °)

**Table:**

O1—C4	1.419 (2)	C5A—C5	1.481 (3)
O1—H1A	0.8200	N1—H1B	0.8600
C2—C1A	1.5001 (18)	C6—H6A	0.9600
C2—C7	1.5224 (18)	C6—H6B	0.9600
C2—C3	1.5535 (19)	C6—H6C	0.9600
C2—H2A	0.9800	C4—C15	1.526 (2)
C7—C12	1.380 (2)	C4—C5	1.537 (2)
C7—C8	1.384 (2)	C12—C11	1.395 (3)
C3—C13	1.530 (2)	C12—H12A	0.9300
C3—C4	1.5573 (19)	C9—C10	1.367 (3)
C3—H3A	0.9800	C9—H9A	0.9300
C1A—C5A	1.380 (2)	C14—H14A	0.9600
C1A—C1	1.387 (2)	C14—H14B	0.9600
O2—C13	1.203 (2)	C14—H14C	0.9600
N2—C1	1.3404 (19)	C5—H5A	0.9700
N2—N1	1.341 (2)	C5—H5B	0.9700
N2—H2B	0.8600	C15—H15A	0.9600
C1—C6	1.476 (2)	C15—H15B	0.9600
C13—C14	1.495 (2)	C15—H15C	0.9600
C8—C9	1.386 (2)	C11—C10	1.364 (4)
C8—H8A	0.9300	C11—H11A	0.9300
C5A—N1	1.336 (2)	C10—H10A	0.9300
			
C4—O1—H1A	109.5	C1—C6—H6C	109.5
C1A—C2—C7	112.15 (11)	H6A—C6—H6C	109.5
C1A—C2—C3	109.00 (11)	H6B—C6—H6C	109.5
C7—C2—C3	110.88 (11)	O1—C4—C15	111.19 (14)
C1A—C2—H2A	108.2	O1—C4—C5	109.66 (14)
C7—C2—H2A	108.2	C15—C4—C5	109.42 (14)
C3—C2—H2A	108.2	O1—C4—C3	106.27 (11)
C12—C7—C8	118.09 (14)	C15—C4—C3	111.47 (14)
C12—C7—C2	121.10 (14)	C5—C4—C3	108.75 (12)
C8—C7—C2	120.80 (12)	C7—C12—C11	120.6 (2)
C13—C3—C2	111.19 (11)	C7—C12—H12A	119.7
C13—C3—C4	111.65 (11)	C11—C12—H12A	119.7
C2—C3—C4	112.95 (12)	C10—C9—C8	119.94 (19)
C13—C3—H3A	106.9	C10—C9—H9A	120.0
C2—C3—H3A	106.9	C8—C9—H9A	120.0
C4—C3—H3A	106.9	C13—C14—H14A	109.5
C5A—C1A—C1	106.91 (13)	C13—C14—H14B	109.5
C5A—C1A—C2	123.23 (14)	H14A—C14—H14B	109.5
C1—C1A—C2	129.81 (13)	C13—C14—H14C	109.5
C1—N2—N1	109.73 (14)	H14A—C14—H14C	109.5
C1—N2—H2B	125.1	H14B—C14—H14C	109.5
N1—N2—H2B	125.1	C5A—C5—C4	109.23 (12)
N2—C1—C1A	106.79 (14)	C5A—C5—H5A	109.8
N2—C1—C6	121.61 (14)	C4—C5—H5A	109.8
C1A—C1—C6	131.60 (13)	C5A—C5—H5B	109.8
O2—C13—C14	120.14 (15)	C4—C5—H5B	109.8
O2—C13—C3	120.00 (15)	H5A—C5—H5B	108.3
C14—C13—C3	119.86 (14)	C4—C15—H15A	109.5
C7—C8—C9	121.12 (16)	C4—C15—H15B	109.5
C7—C8—H8A	119.4	H15A—C15—H15B	109.5
C9—C8—H8A	119.4	C4—C15—H15C	109.5
N1—C5A—C1A	107.79 (15)	H15A—C15—H15C	109.5
N1—C5A—C5	126.19 (14)	H15B—C15—H15C	109.5
C1A—C5A—C5	126.01 (14)	C10—C11—C12	120.22 (19)
C5A—N1—N2	108.77 (13)	C10—C11—H11A	119.9
C5A—N1—H1B	125.6	C12—C11—H11A	119.9
N2—N1—H1B	125.6	C11—C10—C9	120.03 (17)
C1—C6—H6A	109.5	C11—C10—H10A	120.0
C1—C6—H6B	109.5	C9—C10—H10A	120.0
H6A—C6—H6B	109.5		
			
C1A—C2—C7—C12	−137.04 (15)	C1—C1A—C5A—N1	−0.54 (17)
C3—C2—C7—C12	100.85 (16)	C2—C1A—C5A—N1	−178.05 (13)
C1A—C2—C7—C8	42.07 (18)	C1—C1A—C5A—C5	−179.78 (15)
C3—C2—C7—C8	−80.04 (15)	C2—C1A—C5A—C5	2.7 (2)
C1A—C2—C3—C13	170.59 (11)	C1A—C5A—N1—N2	1.10 (19)
C7—C2—C3—C13	−65.48 (14)	C5—C5A—N1—N2	−179.66 (16)
C1A—C2—C3—C4	44.16 (15)	C1—N2—N1—C5A	−1.26 (19)
C7—C2—C3—C4	168.09 (11)	C13—C3—C4—O1	−72.98 (16)
C7—C2—C1A—C5A	−136.73 (14)	C2—C3—C4—O1	53.21 (15)
C3—C2—C1A—C5A	−13.55 (19)	C13—C3—C4—C15	48.32 (18)
C7—C2—C1A—C1	46.4 (2)	C2—C3—C4—C15	174.51 (13)
C3—C2—C1A—C1	169.55 (14)	C13—C3—C4—C5	169.05 (13)
N1—N2—C1—C1A	0.90 (18)	C2—C3—C4—C5	−64.77 (16)
N1—N2—C1—C6	−179.36 (15)	C8—C7—C12—C11	0.7 (3)
C5A—C1A—C1—N2	−0.21 (17)	C2—C7—C12—C11	179.84 (18)
C2—C1A—C1—N2	177.07 (14)	C7—C8—C9—C10	−1.0 (2)
C5A—C1A—C1—C6	−179.92 (16)	N1—C5A—C5—C4	159.56 (16)
C2—C1A—C1—C6	−2.6 (3)	C1A—C5A—C5—C4	−21.3 (2)
C2—C3—C13—O2	125.03 (16)	O1—C4—C5—C5A	−66.44 (16)
C4—C3—C13—O2	−107.83 (16)	C15—C4—C5—C5A	171.35 (15)
C2—C3—C13—C14	−54.72 (17)	C3—C4—C5—C5A	49.37 (18)
C4—C3—C13—C14	72.43 (18)	C7—C12—C11—C10	−1.3 (3)
C12—C7—C8—C9	0.4 (2)	C12—C11—C10—C9	0.7 (4)
C2—C7—C8—C9	−178.70 (14)	C8—C9—C10—C11	0.5 (3)

### Hydrogen-bond geometry (Å, °)

**Table:**

*D*—H···*A*	*D*—H	H···*A*	*D*···*A*	*D*—H···*A*
O1—H1A···Cl^i^	0.82	2.39	3.2110 (14)	176
N2—H2B···Cl^i^	0.86	2.21	3.0620 (14)	171
N1—H1B···Cl^ii^	0.86	2.25	3.0280 (15)	150

Symmetry codes: (i) ; (ii) .
